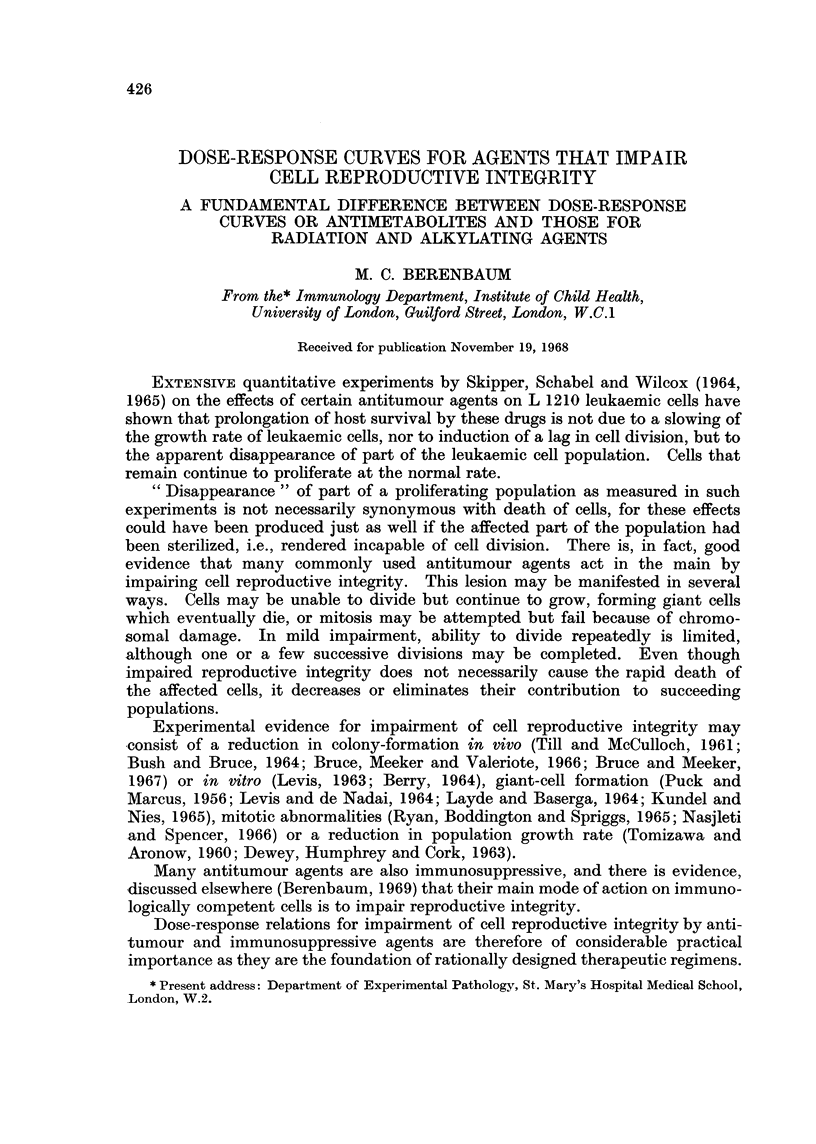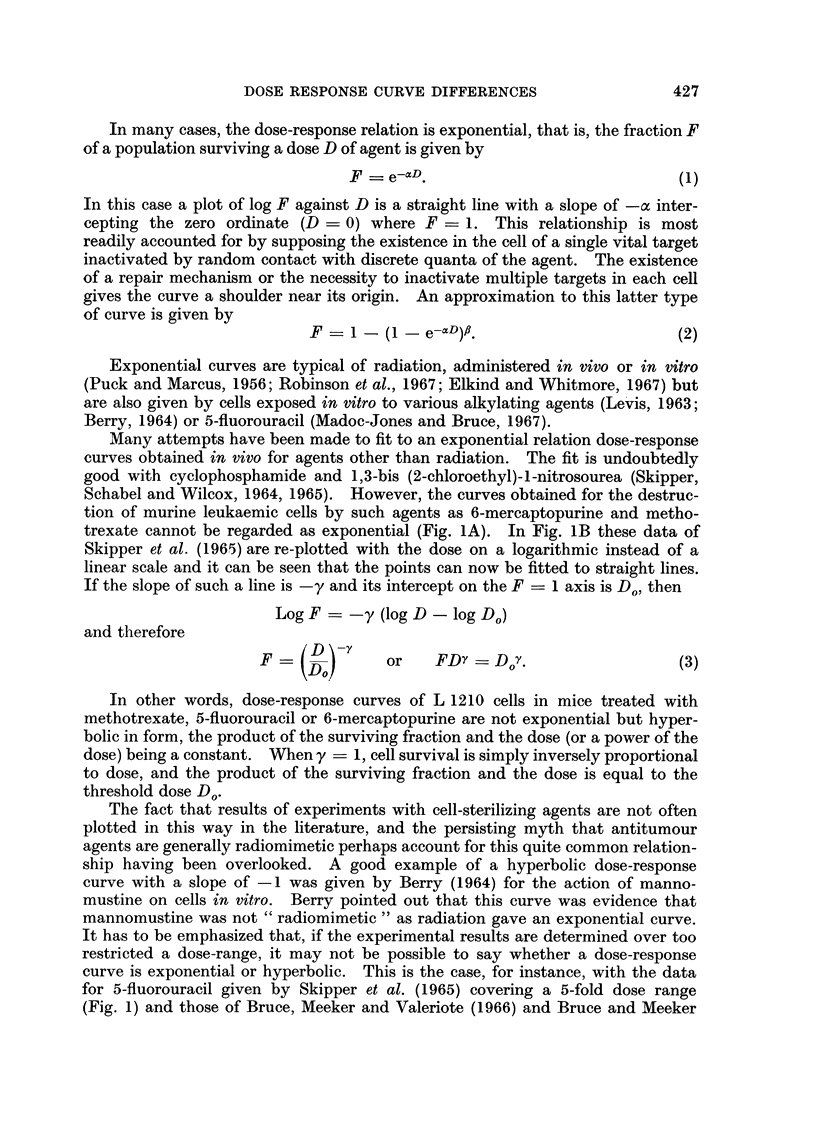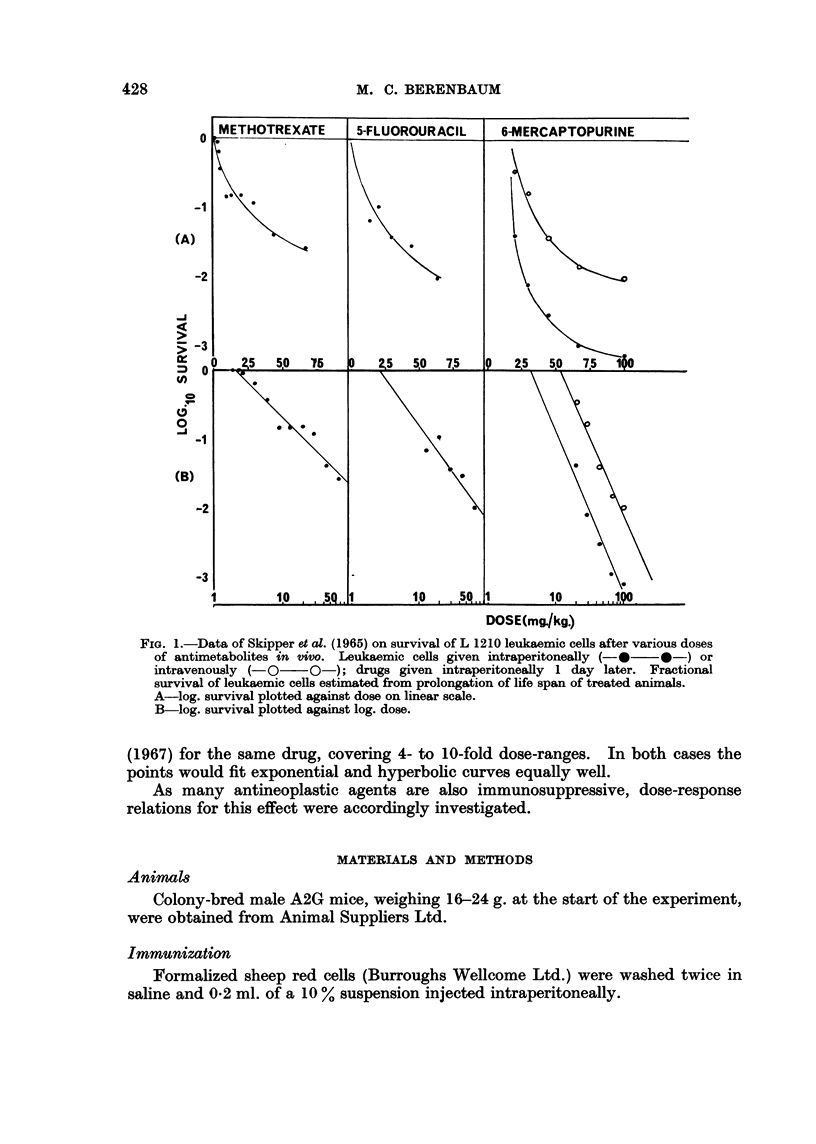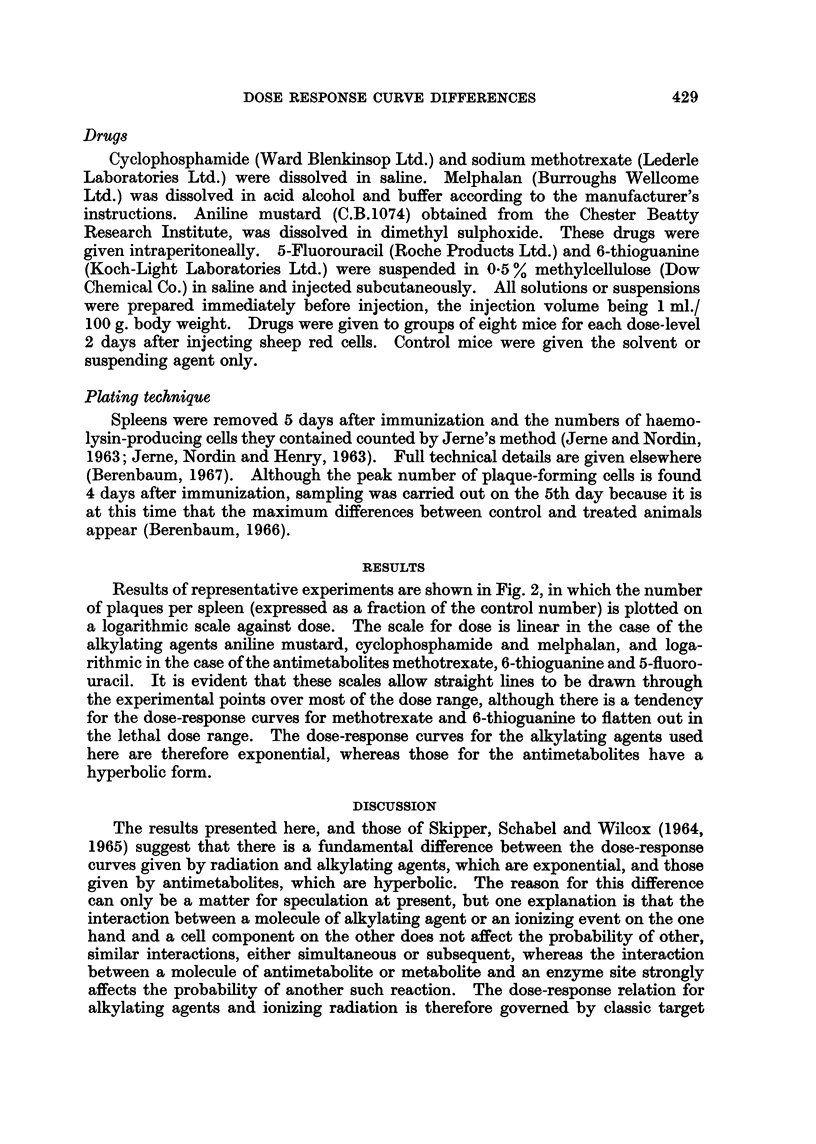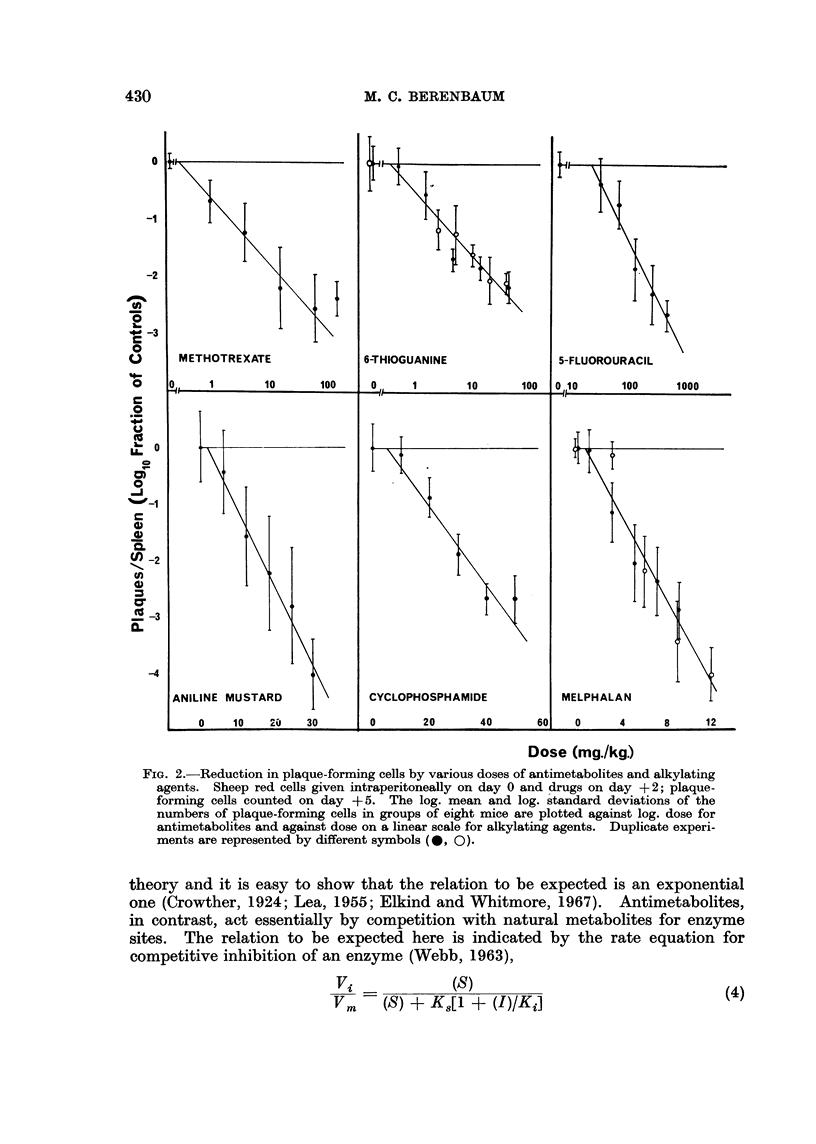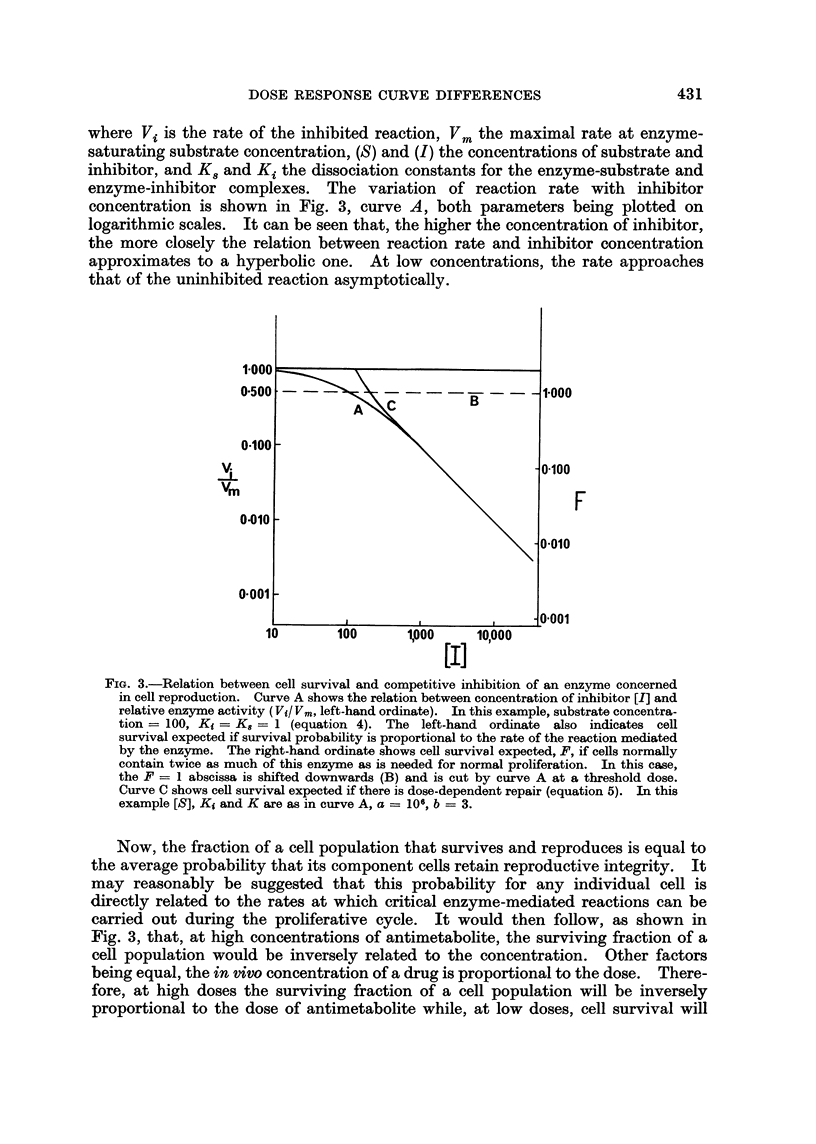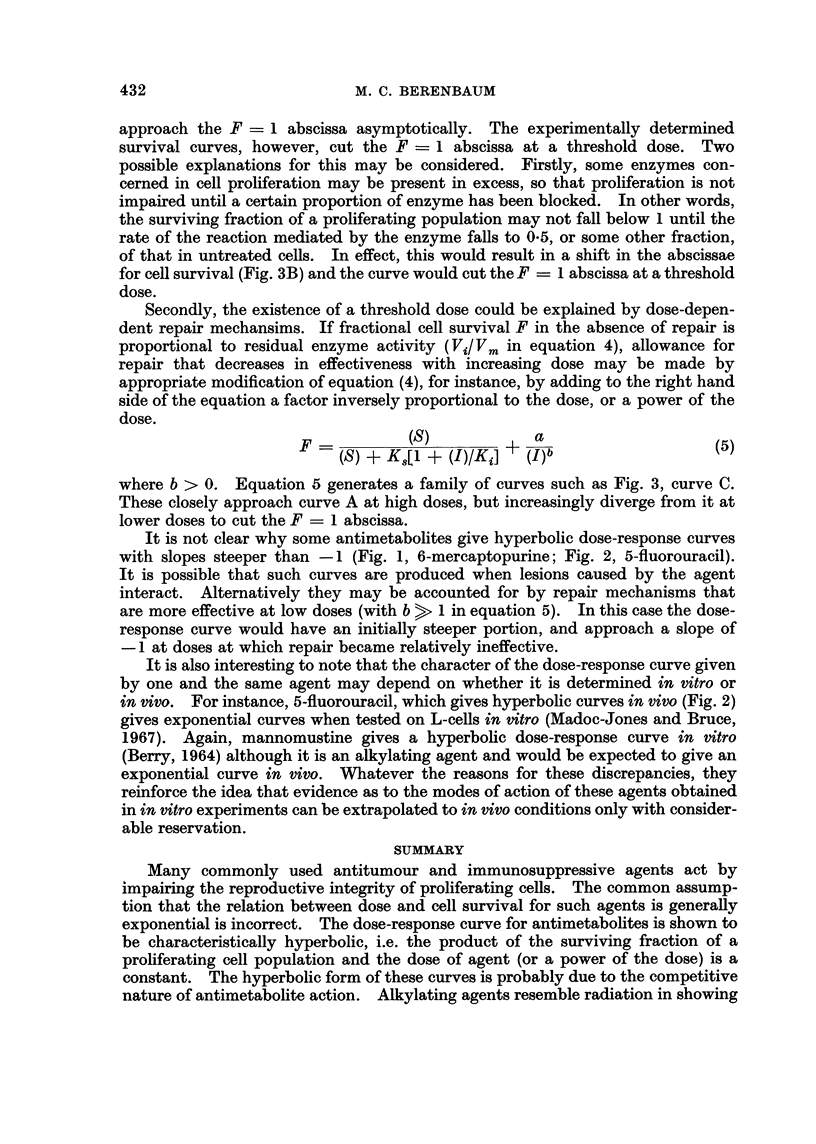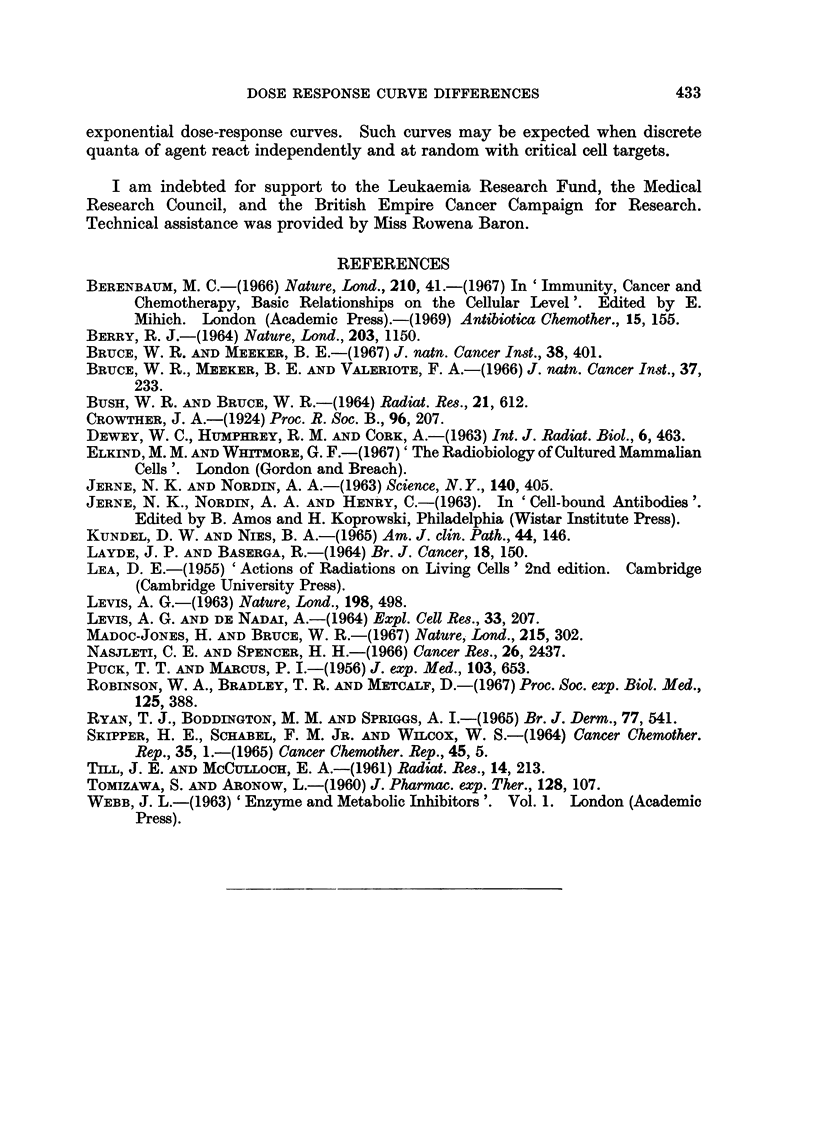# Dose-response curves for agents that impair cell reproductive integrity. A fundamental difference between dose-response curves of antimetabolites and those for radiation and alkylating agents.

**DOI:** 10.1038/bjc.1969.55

**Published:** 1969-06

**Authors:** M. C. Berenbaum


### DOSE-RESPONSE CURVES FOR AGENTS THAT IMPAIR CELL REPRODUCTIVE INTEGRITY

### A FUNDAMENTAL DIFFERENCE BETWEEN DOSE-RESPONSE CURVES OR ANTIMETABOLITES AND THOSE FOR RADIATION AND ALKYLATING AGENTS

M. C. BERENBAUM

*From the* Immunology Department, Institute of Child Health, University of London, Guilford Street, London, W.C.1*

Received for publication November 19, 1968

EXTENSIVE quantitative experiments by Skipper, Schabel and Wilcox (1964, 1965) on the effects of certain antitumour agents on L 1210 leukaemic cells have shown that prolongation of host survival by these drugs is not due to a slowing of the growth rate of leukaemic cells, nor to induction of a lag in cell division, but to the apparent disappearance of part of the leukaemic cell population. Cells that remain continue to proliferate at the normal rate.

" Disappearance " of part of a proliferating population as measured in such experiments is not necessarily synonymous with death of cells, for these effects could have been produced just as well if the affected part of the population had been sterilized, i.e., rendered incapable of cell division. There is, in fact, good evidence that many commonly used antitumour agents act in the main by impairing cell reproductive integrity. This lesion may be manifested in several ways. Cells may be unable to divide but continue to grow, forming giant cells which eventually die, or mitosis may be attempted but fail because of chromosomal damage. In mild impairment, ability to divide repeatedly is limited, although one or a few successive divisions may be completed. Even though impaired reproductive integrity does not necessarily cause the rapid death of the affected cells, it decreases or eliminates their contribution to succeeding populations.

Experimental evidence for impairment of cell reproductive integrity may consist of a reduction in colony-formation *in vivo* (Till and McCulloch, 1961; Bush and Bruce, 1964; Bruce, Meeker and Valeriote, 1966; Bruce and Meeker, 1967) or *in vitro* (Levis, 1963; Berry, 1964), giant-cell formation (Puck and Marcus, 1956; Levis and de Nadai, 1964; Layde and Baserga, 1964; Kundel and Nies, 1965), mitotic abnormalities (Ryan, Boddington and Spriggs, 1965; Nasjleti and Spencer, 1966) or a reduction in population growth rate (Tomizawa and Aronow, 1960; Dewey, Humphrey and Cork, 1963).

Many antitumour agents are also immunosuppressive, and there is evidence, discussed elsewhere (Berenbaum, 1969) that their main mode of action on immunologically competent cells is to impair reproductive integrity.

Dose-response relations for impairment of cell reproductive integrity by antitumour and immunosuppressive agents are therefore of considerable practical importance as they are the foundation of rationally designed therapeutic regimens.

* Present address: Department of Experimental Pathology, St. Mary's Hospital Medical School, London, W.2.

In many cases, the dose-response relation is exponential, that is, the fraction $F$ of a population surviving a dose $D$ of agent is given by

$$F = e^{-\alpha D}. \qquad (1)$$

In this case a plot of log $F$ against $D$ is a straight line with a slope of $-\alpha$ intercepting the zero ordinate ($D = 0$) where $F = 1$. This relationship is most readily accounted for by supposing the existence in the cell of a single vital target inactivated by random contact with discrete quanta of the agent. The existence of a repair mechanism or the necessity to inactivate multiple targets in each cell gives the curve a shoulder near its origin. An approximation to this latter type of curve is given by

$$F = 1 - (1 - e^{-\alpha D})^{\beta}. \qquad (2)$$

Exponential curves are typical of radiation, administered *in vivo* or *in vitro* (Puck and Marcus, 1956; Robinson *et al.*, 1967; Elkind and Whitmore, 1967) but are also given by cells exposed *in vitro* to various alkylating agents (Levis, 1963; Berry, 1964) or 5-fluorouracil (Madoc-Jones and Bruce, 1967).

Many attempts have been made to fit to an exponential relation dose-response curves obtained *in vivo* for agents other than radiation. The fit is undoubtedly good with cyclophosphamide and 1,3-bis (2-chloroethyl)-1-nitrosourea (Skipper, Schabel and Wilcox, 1964, 1965). However, the curves obtained for the destruction of murine leukaemic cells by such agents as 6-mercaptopurine and methotrexate cannot be regarded as exponential (Fig. 1A). In Fig. 1B these data of Skipper *et al.* (1965) are re-plotted with the dose on a logarithmic instead of a linear scale and it can be seen that the points can now be fitted to straight lines. If the slope of such a line is $-\gamma$ and its intercept on the $F = 1$ axis is $D_o$, then

$$\text{Log } F = -\gamma (\log D - \log D_o)$$

and therefore

$$F = \left(\frac{D}{D_o}\right)^{-\gamma} \quad \text{or} \quad FD^{\gamma} = D_o^{\gamma}. \qquad (3)$$

In other words, dose-response curves of L 1210 cells in mice treated with methotrexate, 5-fluorouracil or 6-mercaptopurine are not exponential but hyperbolic in form, the product of the surviving fraction and the dose (or a power of the dose) being a constant. When $\gamma = 1$, cell survival is simply inversely proportional to dose, and the product of the surviving fraction and the dose is equal to the threshold dose $D_o$.

The fact that results of experiments with cell-sterilizing agents are not often plotted in this way in the literature, and the persisting myth that antitumour agents are generally radiomimetic perhaps account for this quite common relationship having been overlooked. A good example of a hyperbolic dose-response curve with a slope of $-1$ was given by Berry (1964) for the action of mannomustine on cells *in vitro*. Berry pointed out that this curve was evidence that mannomustine was not " radiomimetic " as radiation gave an exponential curve. It has to be emphasized that, if the experimental results are determined over too restricted a dose-range, it may not be possible to say whether a dose-response curve is exponential or hyperbolic. This is the case, for instance, with the data for 5-fluorouracil given by Skipper *et al.* (1965) covering a 5-fold dose range (Fig. 1) and those of Bruce, Meeker and Valeriote (1966) and Bruce and Meeker

FIG. 1.—Data of Skipper *et al.* (1965) on survival of L 1210 leukaemic cells after various doses of antimetabolites *in vivo*. Leukaemic cells given intraperitoneally (—●——●—) or intravenously (—○——○—); drugs given intraperitoneally 1 day later. Fractional survival of leukaemic cells estimated from prolongation of life span of treated animals.
A—log. survival plotted against dose on linear scale.
B—log. survival plotted against log. dose.

(1967) for the same drug, covering 4- to 10-fold dose-ranges. In both cases the points would fit exponential and hyperbolic curves equally well.

As many antineoplastic agents are also immunosuppressive, dose-response relations for this effect were accordingly investigated.

### MATERIALS AND METHODS

### *Animals*

Colony-bred male A2G mice, weighing 16–24 g. at the start of the experiment, were obtained from Animal Suppliers Ltd.

### *Immunization*

Formalized sheep red cells (Burroughs Wellcome Ltd.) were washed twice in saline and 0·2 ml. of a 10 % suspension injected intraperitoneally.

*Drugs*

Cyclophosphamide (Ward Blenkinsop Ltd.) and sodium methotrexate (Lederle Laboratories Ltd.) were dissolved in saline. Melphalan (Burroughs Wellcome Ltd.) was dissolved in acid alcohol and buffer according to the manufacturer's instructions. Aniline mustard (C.B.1074) obtained from the Chester Beatty Research Institute, was dissolved in dimethyl sulphoxide. These drugs were given intraperitoneally. 5-Fluorouracil (Roche Products Ltd.) and 6-thioguanine (Koch-Light Laboratories Ltd.) were suspended in 0·5% methylcellulose (Dow Chemical Co.) in saline and injected subcutaneously. All solutions or suspensions were prepared immediately before injection, the injection volume being 1 ml./100 g. body weight. Drugs were given to groups of eight mice for each dose-level 2 days after injecting sheep red cells. Control mice were given the solvent or suspending agent only.

*Plating technique*

Spleens were removed 5 days after immunization and the numbers of haemolysin-producing cells they contained counted by Jerne's method (Jerne and Nordin, 1963; Jerne, Nordin and Henry, 1963). Full technical details are given elsewhere (Berenbaum, 1967). Although the peak number of plaque-forming cells is found 4 days after immunization, sampling was carried out on the 5th day because it is at this time that the maximum differences between control and treated animals appear (Berenbaum, 1966).

### RESULTS

Results of representative experiments are shown in Fig. 2, in which the number of plaques per spleen (expressed as a fraction of the control number) is plotted on a logarithmic scale against dose. The scale for dose is linear in the case of the alkylating agents aniline mustard, cyclophosphamide and melphalan, and logarithmic in the case of the antimetabolites methotrexate, 6-thioguanine and 5-fluorouracil. It is evident that these scales allow straight lines to be drawn through the experimental points over most of the dose range, although there is a tendency for the dose-response curves for methotrexate and 6-thioguanine to flatten out in the lethal dose range. The dose-response curves for the alkylating agents used here are therefore exponential, whereas those for the antimetabolites have a hyperbolic form.

### DISCUSSION

The results presented here, and those of Skipper, Schabel and Wilcox (1964, 1965) suggest that there is a fundamental difference between the dose-response curves given by radiation and alkylating agents, which are exponential, and those given by antimetabolites, which are hyperbolic. The reason for this difference can only be a matter for speculation at present, but one explanation is that the interaction between a molecule of alkylating agent or an ionizing event on the one hand and a cell component on the other does not affect the probability of other, similar interactions, either simultaneous or subsequent, whereas the interaction between a molecule of antimetabolite or metabolite and an enzyme site strongly affects the probability of another such reaction. The dose-response relation for alkylating agents and ionizing radiation is therefore governed by classic target

FIG. 2.—Reduction in plaque-forming cells by various doses of antimetabolites and alkylating agents. Sheep red cells given intraperitoneally on day 0 and drugs on day +2; plaque-forming cells counted on day +5. The log. mean and log. standard deviations of the numbers of plaque-forming cells in groups of eight mice are plotted against log. dose for antimetabolites and against dose on a linear scale for alkylating agents. Duplicate experiments are represented by different symbols (●, ○).

theory and it is easy to show that the relation to be expected is an exponential one (Crowther, 1924; Lea, 1955; Elkind and Whitmore, 1967). Antimetabolites, in contrast, act essentially by competition with natural metabolites for enzyme sites. The relation to be expected here is indicated by the rate equation for competitive inhibition of an enzyme (Webb, 1963),

$$\frac{V_i}{V_m} = \frac{(S)}{(S) + K_s[1 + (I)/K_i]} \qquad (4)$$

where $V_i$ is the rate of the inhibited reaction, $V_m$ the maximal rate at enzyme-saturating substrate concentration, $(S)$ and $(I)$ the concentrations of substrate and inhibitor, and $K_s$ and $K_i$ the dissociation constants for the enzyme-substrate and enzyme-inhibitor complexes. The variation of reaction rate with inhibitor concentration is shown in Fig. 3, curve $A$, both parameters being plotted on logarithmic scales. It can be seen that, the higher the concentration of inhibitor, the more closely the relation between reaction rate and inhibitor concentration approximates to a hyperbolic one. At low concentrations, the rate approaches that of the uninhibited reaction asymptotically.

FIG. 3.—Relation between cell survival and competitive inhibition of an enzyme concerned in cell reproduction. Curve A shows the relation between concentration of inhibitor $[I]$ and relative enzyme activity ($V_i/V_m$, left-hand ordinate). In this example, substrate concentration = 100, $K_i = K_s = 1$ (equation 4). The left-hand ordinate also indicates cell survival expected if survival probability is proportional to the rate of the reaction mediated by the enzyme. The right-hand ordinate shows cell survival expected, $F$, if cells normally contain twice as much of this enzyme as is needed for normal proliferation. In this case, the $F = 1$ abscissa is shifted downwards (B) and is cut by curve A at a threshold dose. Curve C shows cell survival expected if there is dose-dependent repair (equation 5). In this example $[S]$, $K_i$ and $K$ are as in curve A, $a = 10^6$, $b = 3$.

Now, the fraction of a cell population that survives and reproduces is equal to the average probability that its component cells retain reproductive integrity. It may reasonably be suggested that this probability for any individual cell is directly related to the rates at which critical enzyme-mediated reactions can be carried out during the proliferative cycle. It would then follow, as shown in Fig. 3, that, at high concentrations of antimetabolite, the surviving fraction of a cell population would be inversely related to the concentration. Other factors being equal, the *in vivo* concentration of a drug is proportional to the dose. Therefore, at high doses the surviving fraction of a cell population will be inversely proportional to the dose of antimetabolite while, at low doses, cell survival will approach the $F = 1$ abscissa asymptotically. The experimentally determined survival curves, however, cut the $F = 1$ abscissa at a threshold dose. Two possible explanations for this may be considered. Firstly, some enzymes concerned in cell proliferation may be present in excess, so that proliferation is not impaired until a certain proportion of enzyme has been blocked. In other words, the surviving fraction of a proliferating population may not fall below 1 until the rate of the reaction mediated by the enzyme falls to 0·5, or some other fraction, of that in untreated cells. In effect, this would result in a shift in the abscissae for cell survival (Fig. 3B) and the curve would cut the $F = 1$ abscissa at a threshold dose.

Secondly, the existence of a threshold dose could be explained by dose-dependent repair mechansims. If fractional cell survival $F$ in the absence of repair is proportional to residual enzyme activity ($V_i/V_m$ in equation 4), allowance for repair that decreases in effectiveness with increasing dose may be made by appropriate modification of equation (4), for instance, by adding to the right hand side of the equation a factor inversely proportional to the dose, or a power of the dose.

$$F = \frac{(S)}{(S) + K_s[1 + (I)/K_i]} + \frac{a}{(I)^b} \qquad (5)$$

where $b > 0$. Equation 5 generates a family of curves such as Fig. 3, curve C. These closely approach curve A at high doses, but increasingly diverge from it at lower doses to cut the $F = 1$ abscissa.

It is not clear why some antimetabolites give hyperbolic dose-response curves with slopes steeper than $-1$ (Fig. 1, 6-mercaptopurine; Fig. 2, 5-fluorouracil). It is possible that such curves are produced when lesions caused by the agent interact. Alternatively they may be accounted for by repair mechanisms that are more effective at low doses (with $b \gg 1$ in equation 5). In this case the dose-response curve would have an initially steeper portion, and approach a slope of $-1$ at doses at which repair became relatively ineffective.

It is also interesting to note that the character of the dose-response curve given by one and the same agent may depend on whether it is determined *in vitro* or *in vivo*. For instance, 5-fluorouracil, which gives hyperbolic curves *in vivo* (Fig. 2) gives exponential curves when tested on L-cells *in vitro* (Madoc-Jones and Bruce, 1967). Again, mannomustine gives a hyperbolic dose-response curve *in vitro* (Berry, 1964) although it is an alkylating agent and would be expected to give an exponential curve *in vivo*. Whatever the reasons for these discrepancies, they reinforce the idea that evidence as to the modes of action of these agents obtained in *in vitro* experiments can be extrapolated to *in vivo* conditions only with considerable reservation.

### SUMMARY

Many commonly used antitumour and immunosuppressive agents act by impairing the reproductive integrity of proliferating cells. The common assumption that the relation between dose and cell survival for such agents is generally exponential is incorrect. The dose-response curve for antimetabolites is shown to be characteristically hyperbolic, i.e. the product of the surviving fraction of a proliferating cell population and the dose of agent (or a power of the dose) is a constant. The hyperbolic form of these curves is probably due to the competitive nature of antimetabolite action. Alkylating agents resemble radiation in showing exponential dose-response curves. Such curves may be expected when discrete quanta of agent react independently and at random with critical cell targets.

I am indebted for support to the Leukaemia Research Fund, the Medical Research Council, and the British Empire Cancer Campaign for Research. Technical assistance was provided by Miss Rowena Baron.

### REFERENCES

BERENBAUM, M. C.—(1966) *Nature, Lond.*, **210**, 41.—(1967) In ' Immunity, Cancer and Chemotherapy, Basic Relationships on the Cellular Level'. Edited by E. Mihich. London (Academic Press).—(1969) *Antibiotica Chemother.*, **15**, 155.
BERRY, R. J.—(1964) *Nature, Lond.*, **203**, 1150.
BRUCE, W. R. AND MEEKER, B. E.—(1967) *J. natn. Cancer Inst.*, **38**, 401.
BRUCE, W. R., MEEKER, B. E. AND VALERIOTE, F. A.—(1966) *J. natn. Cancer Inst.*, **37**, 233.
BUSH, W. R. AND BRUCE, W. R.—(1964) *Radiat. Res.*, **21**, 612.
CROWTHER, J. A.—(1924) *Proc. R. Soc.* B., **96**, 207.
DEWEY, W. C., HUMPHREY, R. M. AND CORK, A.—(1963) *Int. J. Radiat. Biol.*, **6**, 463.
ELKIND, M. M. AND WHITMORE, G. F.—(1967) ' The Radiobiology of Cultured Mammalian Cells '. London (Gordon and Breach).
JERNE, N. K. AND NORDIN, A. A.—(1963) *Science, N.Y.*, **140**, 405.
JERNE, N. K., NORDIN, A. A. AND HENRY, C.—(1963). In ' Cell-bound Antibodies '. Edited by B. Amos and H. Koprowski, Philadelphia (Wistar Institute Press).
KUNDEL, D. W. AND NIES, B. A.—(1965) *Am. J. clin. Path.*, **44**, 146.
LAYDE, J. P. AND BASERGA, R.—(1964) *Br. J. Cancer*, **18**, 150.
LEA, D. E.—(1955) ' Actions of Radiations on Living Cells ' 2nd edition. Cambridge (Cambridge University Press).
LEVIS, A. G.—(1963) *Nature, Lond.*, **198**, 498.
LEVIS, A. G. AND DE NADAI, A.—(1964) *Expl. Cell Res.*, **33**, 207.
MADOC-JONES, H. AND BRUCE, W. R.—(1967) *Nature, Lond.*, **215**, 302.
NASJLETI, C. E. AND SPENCER, H. H.—(1966) *Cancer Res.*, **26**, 2437.
PUCK, T. T. AND MARCUS, P. I.—(1956) *J. exp. Med.*, **103**, 653.
ROBINSON, W. A., BRADLEY, T. R. AND METCALF, D.—(1967) *Proc. Soc. exp. Biol. Med.*, **125**, 388.
RYAN, T. J., BODDINGTON, M. M. AND SPRIGGS, A. I.—(1965) *Br. J. Derm.*, **77**, 541.
SKIPPER, H. E., SCHABEL, F. M. JR. AND WILCOX, W. S.—(1964) *Cancer Chemother. Rep.*, **35**, 1.—(1965) *Cancer Chemother. Rep.*, **45**, 5.
TILL, J. E. AND MCCULLOCH, E. A.—(1961) *Radiat. Res.*, **14**, 213.
TOMIZAWA, S. AND ARONOW, L.—(1960) *J. Pharmac. exp. Ther.*, **128**, 107.
WEBB, J. L.—(1963) ' Enzyme and Metabolic Inhibitors '. Vol. 1. London (Academic Press).